# Risk Factors for Impaired Glucose Metabolism in Transfusion-Dependent Patients with β-Thalassemia: A Single-Center Retrospective Observational Study

**DOI:** 10.3390/hematolrep17010006

**Published:** 2025-01-30

**Authors:** Theodora Maria Venou, Filippos Kyriakidis, Fani Barmpageorgopoulou, Stamatia Theodoridou, Athanasios Vyzantiadis, Philippos Klonizakis, Eleni Gavriilaki, Efthymia Vlachaki

**Affiliations:** 1 Adult Thalassemia Unit, 2nd Department of Internal Medicine, Aristotle University of Thessaloniki, Hippokration General Hospital, 54642 Thessaloniki, Greece; philklon@auth.gr; 2 Department of Medicine, School of Health Sciences, Aristotle University of Thessaloniki, 54124 Thessaloniki, Greece; 3 Hemoglobinopathies Prevention Unit, Hippokration General Hospital, 41221 Thessaloniki, Greeceavyza@auth.gr (A.V.); 4 2nd Propedeutical Department of Internal Medicine, Aristotle University of Thessaloniki, Hippokration General Hospital, 54124 Thessaloniki, Greece; gavriiel@auth.gr

**Keywords:** β-thalassemia, endocrinopathies, impaired glucose tolerance, diabetes mellitus

## Abstract

Background/Objectives: B-thalassemia is a genetic disorder that leads to reduced or absent β-globin chains, often resulting in endocrine abnormalities due to iron overload, chronic anemia, and hypoxia. This study investigates the prevalence and risk factors for glucose metabolism disturbances in transfusion-dependent β-thalassemia (TDT) patients, focusing on pancreatic iron overload and its association with other iron biomarkers. Methods: We studied two groups of TDT patients (2018–2022) at Hippokration General Hospital: Group 1 (no glucose metabolism impairment, n = 46) and Group 2 (with impaired glucose tolerance or diabetes mellitus, n = 18). Patients were assessed for factors contributing to glucose disturbances, and laboratory data were analyzed. Type 2 diabetes was diagnosed per American Diabetes Association criteria, and impaired glucose tolerance was defined by OGTT results. A multivariate logistic regression identified potential independent risk factors. In a subset of patients on iron chelation therapy, we examined the relationship between pancreatic, liver, and heart iron overload (T2* MRI) and glucose/ferritin levels. Results: Age and elevated serum GGT levels were significantly associated with impaired glucose metabolism (*p* = 0.02). Beta-blocker use was correlated with glucose disturbances (*p* = 0.02), but multivariate analysis revealed no significant independent risk factors. A significant relationship was found between pancreatic and heart iron overload (r = 0.45, *p* = 0.04). Conclusions: Elevated GGT levels suggest that oxidative stress and liver dysfunction play a key role in glucose metabolism disturbances. Pancreatic MRI T2* may help predict heart iron overload. Further research is needed to identify reliable biomarkers for glucose regulation in TDT.

## 1. Introduction

B-thalassemia is a genetic blood disorder characterized by a reduction or absence of β-globin chains, leading to anemia [1]. The imbalance between α- and β-globin chains damages red blood cells, causing ineffective erythropoiesis, tissue hypoxia, and iron overload due to both increased absorption and transfusions [2]. This iron overload primarily affects the liver, heart, and endocrine organs, leading to serious complications [3].

In the past, β-thalassemia was classified into three types: major, intermediate, and the trait of β-thalassemia [4]. Currently, β-thalassemia is classified as transfusion-dependent or non-transfusion-dependent, depending on whether a strict regimen of regular transfusions is required. The transfusion-dependent form (TDT) requires lifelong monitoring and close follow-up, ideally in specialized transfusion units staffed by multidisciplinary teams. Proper management of this condition is essential for maintaining the patient’s health and well-being [5].

Treatment includes regular red blood cell transfusions and iron chelation therapy to manage iron levels, using agents like deferoxamine, deferiprone, and deferasirox [6]. Despite advances, complications such as diabetes mellitus (DM), hypothyroidism, osteoporosis, and hypogonadism remain common due to iron overload, especially in long-living patients. These conditions impact quality of life, posing challenges for early diagnosis and management of glucose metabolism disorders in thalassemia patients [1,7].

Accurately estimating iron overload is essential for adjusting iron chelation therapy in thalassemia patients. While serum ferritin is commonly used to assess iron overload, it can be influenced by factors like inflammation and liver dysfunction [7]. MRI provides a non-invasive, reproducible method for measuring iron deposition in the liver, heart, and other organs. Liver MRI estimates liver iron concentration (LIC) and correlates well with biopsy results, with a target range of 2–7 mg/g of dry weight to prevent complications. Cardiac MRI detects heart iron overload before heart failure develops. Pancreatic MRI, though not routine, can detect β-cell dysfunction linked to glucose metabolism impairment, which is crucial for preventing diabetes [7]. Additionally, the ISSI-2 index, combined with MRI, can identify high-risk patients early, potentially replacing the need for oral glucose tolerance tests (OGTT) in some cases [8,9,10,11].

Diabetes mellitus (DM) refers to a group of metabolic disorders characterized by impaired carbohydrate, lipid, and protein metabolism. The term “diabetes” originates from the Greek word “diabaino” (διαβαίνω), meaning “to pass through,” and “mellitus” was added by Thomas Willis in the 17th century to describe the sweet taste of diabetic urine [12]. Diabetes, first noted by ancient civilizations, affects 9.3% of the global population as of 2019, a figure projected to rise to 10.9% by 2045 [13]. According to the classification of WHO (2019), diabetes mellitus (DM) can be divided as follows: (a) type 1 diabetes mellitus, (b) type 2 diabetes mellitus, (c) hybrid forms, (d) other specific types (monogenic diabetes, diseases of the exocrine pancreas, secondary to other exocrine disorders, related to drug use or infection, present in the context of a genetic syndrome or another form of immune-mediated DM), and finally, (e) hyperglycemia during pregnancy (gestational DM) [14]. Type 1 involves autoimmune destruction of pancreatic β-cells in younger individuals, while type 2, seen in older adults, stems from decreased insulin sensitivity and secretion. Thalassemia-related diabetes belongs to the category of DM caused by underlying clinical conditions, such as iron overload due to frequent transfusions [8,15].

Diabetes mellitus in β-thalassemia patients, driven by iron overload, involves distinct pathophysiological mechanisms [7]. Initially, insulin resistance is thought to result from iron accumulation in the liver and muscles, which increases insulin production. However, as iron deposits in the pancreas, β-cell dysfunction arises, leading to reduced insulin production, sometimes preceding impaired glucose tolerance (IGT) [7,16]. Hepatitis C infection (HCV) further disrupts glucose metabolism disturbances by enhancing pancreatic iron deposition [17].

Several factors are associated with a higher risk of developing IGT or diabetes, including advanced age [18,19,20], splenectomy [19,21], body weight [22], the necessary number of transfused packed red blood cells units [20], and elevated serum ferritin levels [18,19,20,22,23,24]. Although chronic iron overload plays a pivotal role, acute serum ferritin levels may not always predict the onset of diabetes [25]. Additional contributors include zinc deficiency [26], genetic factors like the IVS2-745 mutation, and autoimmune mechanisms [7,25,27,28], all of which can influence the risk of diabetes in thalassemia patients.

Diagnosing impaired glucose metabolism in β-thalassemia patients is challenging due to the unique pathophysiology associated with thalassemia-induced diabetes [6]. While fasting glucose testing is recommended biannually from early childhood, it may fail to detect cases of impaired glucose tolerance (IGT). The oral glucose tolerance test (OGTT) offers greater sensitivity but is also time-consuming and subject to variability. As a result, some guidelines suggest using fasting and postprandial glucose levels as alternative markers.

International standards recommend initiating fasting glucose testing at age 5 or 10 and continuing it every six months. OGTT is advised if fasting glucose exceeds 110 mg/dL. Additionally, the “Standards of Care Guidelines for Thalassemia, 2012” and “ICET-A Standards of Care 2013” recommend a 2 h, 75 g OGTT at ages 10, 12, 14, and 16, followed by annual testing thereafter [7,8]. An optimal diagnostic approach combines the 2 h OGTT with measurements of insulin secretion [8]. The latest diagnostic criteria for diabetes mellitus include (1) HbA1c ≥ 6.5%, (2) fasting glucose ≥ 126 mg/dL, (3) 2 h plasma glucose ≥ 200 mg/dL (≥11.1 mmol/L) during OGTT, or (4) random plasma glucose ≥ 200 mg/dL [5].

Ongoing monitoring and personalized iron chelation therapies are essential for managing glucose metabolism disturbances in this population. HbA1c, a widely used marker for long-term glucose control, is less reliable in thalassemia patients due to hemoglobinopathies caused by the reduced life span of erythrocytes [29,30]. Alternative markers, such as fructosamine and glycated albumin, provide options for short-term glycemic monitoring, although their clinical utility remains a topic of debate [18,31,32].

Continuous glucose monitoring systems (CGMS) offer valuable insights into daily glucose fluctuations, but clear guidelines for their use in thalassemia patients have not yet been established [7,8]. Further research is needed to identify the most effective methods for the early detection and monitoring of glucose metabolism disturbances in β-thalassemia patients.

Proper adherence to iron chelation therapy is crucial for preventing and managing glucose metabolism disturbances in TDT patients, as iron overload significantly contributes to both insulin resistance and pancreatic β-cell dysfunction. Intensified chelation therapy has demonstrated improvements in glucose metabolism by reducing iron overload in the liver and heart, though its effectiveness in lowering pancreatic iron remains limited [33,34].

Combination therapy, particularly using deferoxamine (DFO) and deferiprone, has proven more effective than monotherapy in reducing pancreatic iron and improving β-cell function, insulin resistance, and glucose intolerance [18,33,35]. While oral antidiabetic agents such as metformin, glibenclamide, and acarbose are sometimes employed, research on their efficacy in managing thalassemia-related diabetes is limited [7]. Insulin therapy is frequently required when glucose disturbances progress to overt diabetes [8,18].

This retrospective, observational study aimed to investigate the prevalence and contributing factors of glucose metabolism disturbances in transfusion-dependent β-thalassemia patients in northern Greece. We evaluated the impact of various factors, including age, gender, specific medications, type of iron chelation, blood type, genotype, and coexisting conditions (hypothyroidism, splenectomy, HCV infection), as well as cardiac and hepatic iron levels (measured via MRI T2* and serum ferritin), on the likelihood of impaired glucose metabolism in these patients.

Furthermore, we examined whether specific laboratory biomarkers are significantly altered in thalassemia patients with diabetes mellitus or impaired glucose tolerance (IGT). Lastly, we explored the relationship between pancreatic iron overload and overall iron burden, as reflected by other iron overload indicators such as heart and liver MRI T2* values and serum ferritin levels.

## 2. Materials and Methods

The study population included consecutive patients with transfusion-dependent β-thalassemia (TDT) who received regular transfusions at the Adult Thalassemia Unit, 2nd Department of Internal Medicine, Hippokration General Hospital, between 2018 and 2022. Eligibility criteria required a confirmed diagnosis of transfusion-dependent β-thalassemia, age over 20 years, regular transfusions of packed red blood cells (every 2–4 weeks), systemic iron chelation therapy, no history of type 1 diabetes, and willingness to provide written informed consent.

Exclusion criteria included non-transfusion-dependent thalassemia, a diagnosis or family history of type 1 diabetes mellitus, and pregnancy. For non-diabetic participants, an essential requirement was undergoing a 2 h oral glucose tolerance test during the study period. The presence of other endocrinopathies did not disqualify participants. Pregnant women were excluded to eliminate potential confounding factors from gestational diabetes mellitus.

All participants provided informed consent. The study was approved by the Ethics Committee of Aristotle University of Thessaloniki (approval code: 180-2023/approval date: 21 June 2023) and conducted in accordance with the Declaration of Helsinki. The researchers declare no conflicts of interest or funding sources.

Diabetes mellitus was diagnosed based on the American Diabetes Association guidelines, defined by fasting plasma glucose (FPG) levels > 126 mg/dL or a 120 min glucose value > 200 mg/dL. Impaired glucose tolerance was diagnosed using the oral glucose tolerance test (OGTT), during which venous plasma samples were collected at 0, 30, 60, 120, and 180 min after the consumption of an 8-ounce solution containing 75 g of glucose. A 2 h blood glucose level between 140 and 199 mg/dL was classified as indicative of IGT [5].

The following factors were extracted from patients’ medical records: medications affecting glucose metabolism (e.g., corticosteroids, hormone replacement therapy, β-blockers), history of HCV infection, total splenectomy, age at the time of data collection, gender, type of chelation therapy (over the past year), genotype, MRI T2* values for the pancreas, heart, and liver, cardiac ejection fraction, annual transfused blood volume, and mean body weight. Additionally, laboratory test results from the hospital’s system, averaged over the study period, were recorded and analyzed. These included hemoglobin, absolute blood cell count, serum ferritin levels, hepatic function biomarkers (creatine phosphokinase (CPK), alkaline phosphatase (ALP), transaminases), hemolysis markers (lactate dehydrogenase (LDH), total bilirubin, direct bilirubin), amylase, gamma-glutamyl transferase (GGT), C-reactive protein (CRP), and kidney function tests (serum urea and creatinine).

MRI imaging was used to obtain quantitative multi-echo sequences for relaxation time measurement (T2* in ms). T2* values were calculated by focusing on the parenchyma of the organs using 12 echo times and a single-exponential recovery model. For the liver, liver iron concentration (LIC) was expressed in μmol/g of dry liver weight, calculated using the formula from Henninger et al. (2015) [Fe = 0.024 × R2* + 0.277], where R2* is derived from T2* values (R2* = 1000/T2*).

In a subset of transfusion-dependent β-thalassemia patients undergoing iron chelation therapy, we investigated the relationship between pancreatic iron overload (MRI T2*), liver and heart overload (MRI T2*), and serum glucose and ferritin levels. Iron overload severity was categorized as mild, moderate, or severe based on MRI T2* and serum ferritin levels (Table 1).

Statistical analysis was performed using SPSS (version 29.0.1.0), with a significance level set at 0.05. The normality of all numeric variables was initially assessed using the Shapiro–Wilk test, and the results are summarized in Table 2. A *p*-value greater than 0.05 indicated normally distributed data. Variables such as age, hemoglobin, neutrophil and lymphocyte blood counts, serum urea and creatinine, ejection fraction, and cardiac iron concentration (T2* MRI) were found to be normally distributed (*p* > 0.05). All other variables were non-normally distributed (*p* < 0.05). The normality of distributions was further confirmed through graphical methods.

Patients were divided into two groups based on their glycemic status: Group 1 (patients without diabetes mellitus or impaired glucose tolerance) and Group 2 (patients with diabetes mellitus or impaired glucose tolerance). Comparisons between the two groups were conducted using the unpaired Student’s *t*-test for normally distributed variables and the Mann–Whitney U test for non-normally distributed variables. Levene’s test was performed to check for equality of variances in non-normally distributed variables. The chi-square (χ^2^) test was used to evaluate associations between categorical variables.

To examine the impact of independent variables on the development of impaired glucose metabolism, a multivariate logistic regression analysis was conducted, as the dependent variable is binary (presence or absence of impaired glucose metabolism). Initially, a univariate analysis was performed for each independent variable (numeric or categorical) to identify candidates for inclusion in the multivariate model. Variables with a *p*-value < 0.2 were included for further analysis.

Additionally, Pearson’s correlation coefficient was calculated to evaluate the relationship between liver, pancreas, and heart MRI T2* values and serum glucose and ferritin levels. This analysis was conducted on a subset of patients with available pancreatic MRI T2* data. Patients were further categorized into two groups based on their history of splenectomy, and Mann–Whitney tests were performed to explore associations between splenectomy status and other variables. Finally, the subset was divided into two groups based on ferritin levels, with a cut-off value of 1000 μg/L.

## 3. Results

In this single-center retrospective observational study, we examined the prevalence and risk factors for diabetes mellitus (DM) and impaired glucose tolerance (IGT) in 64 patients with transfusion-dependent β-thalassemia [29 males (45.3%) and 35 females (54.6%)]. Of the 140 patients who received regular transfusions during the study period, only 56 had recently undergone an oral glucose tolerance test (OGTT). No patients were excluded due to pregnancy or a family history of type 1 diabetes.

Patients were divided into two groups based on their glycemic status: Group 1 (without DM or IGT) included 46 patients (71.9%), comprising 20 males and 26 females, while Group 2 (with DM or IGT) included 18 patients (28.1%), with 9 males and 9 females (Table 2). No significant association was observed between gender and the occurrence of impaired glucose metabolism (*p* = 0.63).

Table 3 provides descriptive statistics for the numerical variables in our dataset. For variables following a normal distribution, the results are presented as the mean and standard deviation (mean, SD). For non-normally distributed variables, the data are expressed as the median and interquartile range (median, IQR).

**Figure 1:**
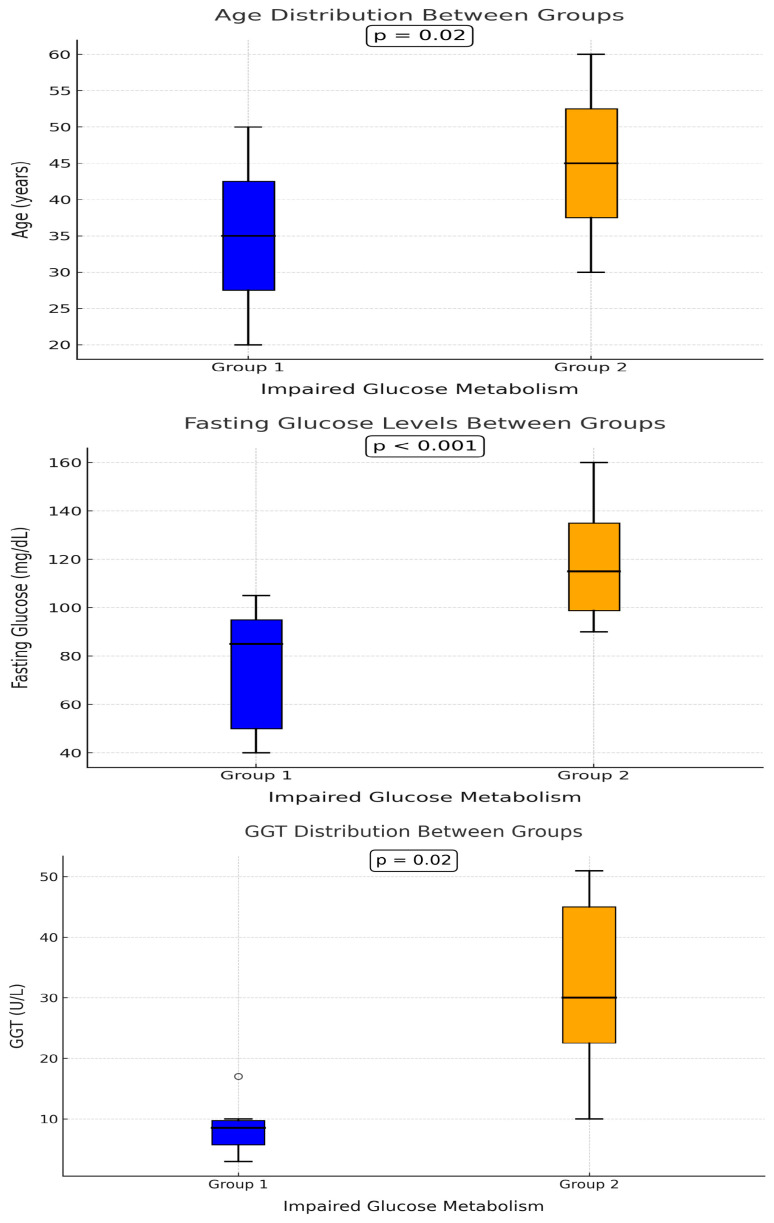
Comparison of age, fasting glucose levels, and GGT distribution between Group 1 and Group 2, highlighting differences in impaired glucose metabolism. Statistical significance is indicated with *p*-values. Group 1 is represented in blue, and Group 2 in orange.

The mean age was significantly lower in Group 1 compared to Group 2, with a statistically significant difference between the two groups [mean age (SD)—Group 1: 39 (9), Group 2: 45.4 (10), *p* = 0.002] (Figure 1). As expected, fasting glucose levels were significantly higher in Group 2 than in Group 1 [median glucose (IQR)—Group 1: 80 (10), Group 2: 104 (42), *p* < 0.01] (Figure 1).

Serum GGT levels also showed a significant difference, with Group 2 having significantly higher median values compared to Group 1 [median GGT (IQR)—Group 1: 14 (8), Group 2: 22 (18), *p* = 0.02] (Figure 1). However, GGT levels did not exhibit a statistically significant correlation with serum ferritin levels (*p* = 0.64, >0.05). Among patients in Group 2, no significant difference was found between those with DM and those with IGT regarding age and serum GGT levels (*p* = 0.46 and *p* = 0.57, respectively, >0.05).

**Figure 2:**
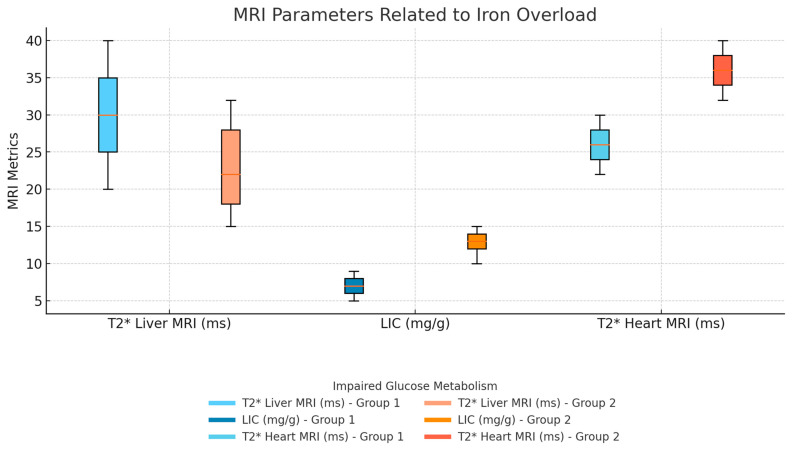
Heart and liver MRI T2* values and liver iron concentration (LIC) between groups. No statistically significant difference was found in heart and liver MRI T2* values, as well as liver iron concentration (LIC) between Group 1 and Group 2 (*p* > 0.05).

No significant differences were observed between the two groups for the other investigated parameters, including body weight, serum hemoglobin, white blood cell and monocyte counts, platelets, serum urea and creatinine, hemolysis markers (LDH, total, direct, and indirect bilirubin), transaminases (ALT, AST), CRP, serum amylase, liver and heart MRI T2* values, LIC, serum ferritin levels, and ejection fraction (*p* > 0.05) (Figure 2). Additionally, no patient had an ejection fraction below 50%.

Regarding the use of medications potentially contributing to impaired glucose metabolism, beta-blocker administration was significantly associated with the development of glucose metabolism disturbances (*p* = 0.02 < 0.05). Notably, approximately 40% of patients in Group 2 were treated with beta-blockers, compared to only 13% in Group 1. However, no statistically significant differences were observed between the two groups in the use of hormone replacement therapy, corticosteroids, or zinc supplementation (Table 4).

**Figure 3:**
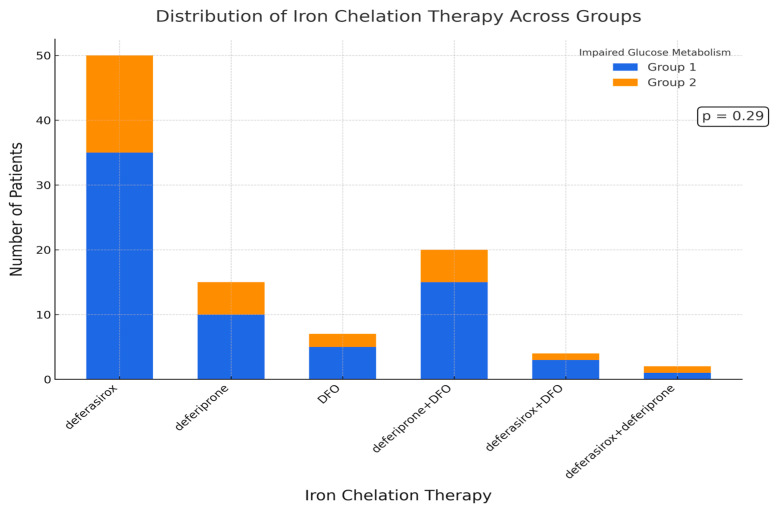
Chelation therapy types in Group 1 and Group 2. The majority of patients in both groups were treated with deferasirox, followed by a combination of deferoxamine (DFO) and deferiprone. No significant relationship was found between the type of iron chelation therapy and the occurrence of impaired glucose metabolism (*p* > 0.05).

In terms of iron chelation therapy, most patients were treated with deferasirox (37/64, 57.5%), followed by a combination of deferoxamine (DFO) and deferiprone (15/64, 23.4%). No statistically significant differences were observed between the two groups regarding the type of chelation therapy (*p* = 0.29) (Figure 3).

With respect to antidiabetic treatment, two patients with diagnosed diabetes mellitus were receiving insulin therapy, consisting of a combination of fast-acting and basal insulin. The remaining patients were managed with oral antidiabetic agents. These included monotherapy with metformin (2 patients), a combination of metformin with other oral agents (2 patients), and other oral agents such as DPP-4 inhibitors, SGLT-2 inhibitors, and GLP-1 receptor agonists (2 patients).

Most patients in both groups were euthyroid [Group 1: 39/46 (84.7%), Group 2: 12/18 (66.6%)] and had no positive history of HCV infection [Group 1: 38/46 (82.6%), Group 2: 13/18 (72.2%)], with no statistically significant difference between the groups (*p* > 0.05). In Group 1, the majority of patients had not undergone splenectomy (18/46, 28%), while in Group 2, most patients had undergone splenectomy (10/18, 55.5%). However, splenectomy status was not significantly associated with the presence of diabetes mellitus or impaired glucose tolerance (*p* = 0.23) (Table 5).

It is worth noting that no patients had a positive RNA PCR test for HCV, and none were undergoing treatment for active HCV infection.

**Figure 4:**
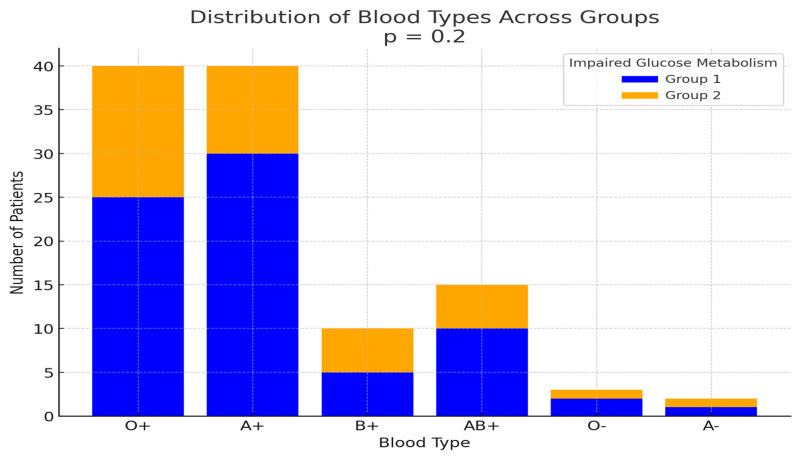
Blood type distribution according to the ABO rhesus system in Group 1 and Group 2. This graph illustrates the distribution of blood types in both groups. In Group 1, the most prevalent blood type was A+, followed by O+. In Group 2, O+ was the most prevalent blood type.

The most prevalent blood type in Group 1, according to the ABO Rh system, was A+ (19/46, 41.3%), followed by O+ (18/46, 39.1%), with a small difference between the two. In contrast, the most common blood type in Group 2 was O+ (11/18, 61.1%). However, no significant association was observed between blood type and the development of impaired glucose metabolism (*p* = 0.2) (Figure 4).

Genotype information was available for 39 out of 64 patients. The most common genotype was IVS1:110/IVS1:110, which was identified in approximately one-third of the patients (13/39, 33.3%). Among these, five out of thirteen patients exhibited impaired glucose metabolism. Additionally, of the three patients with at least one IVS2-745 allele, two displayed glucose metabolism disturbances (one with diabetes mellitus and one with an abnormal OGTT). However, no statistically significant difference in genotype distribution was found between the two groups (*p* = 0.12, >0.05).

Based on the results from the separate univariate logistic regression analyses, age, heart MRI T2*, beta-blocker treatment, and serum AST, ALT, and GGT levels were included in the multivariate logistic regression model. Serum glucose was excluded from the analysis. The overall model was statistically significant when compared to the null model (χ^2^(6) = 15.6, *p* = 0.016). However, the multivariate regression analysis revealed that none of the investigated variables had a significant impact on the development of glucose metabolism disturbances when controlling for other predictors (*p* > 0.05).

Interestingly, the risk of developing impaired glucose metabolism was 2.7 times higher in patients who receiving beta-blockers compared to those who did not; however, this association was not statistically significant (*p* = 0.18, 95% CI: 0.63–1.7). Table 6 and Table 7 summarize the findings from the univariate and multivariate logistic regression analyses.

Finally, we analyzed a subset of 20 patients with available data on pancreatic MRI T2* (Table 8). All investigated parameters were non-normally distributed as indicated by the Shapiro–Wilk test (*p* < 0.05). A statistically significant positive linear relationship was observed between pancreatic MRI T2* and heart MRI T2* (Pearson’s coefficient: 0.45, *p* = 0.04), indicating a moderate correlation between these two variables. However, no other statistically significant correlations were found between MRI T2* values and any other variables (*p* > 0.05). Additionally, no significant difference in pancreatic MRI T2* values was detected between splenectomized and non-splenectomized patients (*p* > 0.05).

All 20 patients in the subset had normal heart and liver MRI T2* values, with heart MRI T2* > 20 ms and liver MRI T2* > 6.3 ms, based on the applied criteria. Notably, none of the patients had ferritin levels exceeding 2000 μg/L. Furthermore, there was no statistically significant difference in pancreatic MRI T2* values between patients with normal ferritin levels and those with mildly elevated ferritin levels, using a cut-off value of 1000 μg/L (*p* > 0.05).

## 4. Discussion

The present retrospective observational study aligns with findings from previous research. It confirms that impaired glucose metabolism is associated with age in patients with transfusion-dependent thalassemia (TDT), mirroring trends observed in the general population. The study also identified significantly higher GGT levels in patients with DM and IGT, underscoring the role of oxidative stress and liver dysfunction in the development of glucose metabolism disturbances in TDT patients. Furthermore, this is the first study to explore the impact of beta-blocker use on glucose metabolism disturbances in TDT patients. Lastly, the study demonstrated a significant relationship between pancreatic and heart MRI T2* values, suggesting a potential link between pancreatic iron overload and total body iron burden.

In this study, patients with impaired glucose metabolism were significantly older than those with normal glucose levels. Consistent with previous research, age was strongly correlated with impaired glucose metabolism [18,20]. For instance, a study by El-Samahy et al. reported a higher risk of impaired fasting glucose (IFG) and diabetes mellitus (DM) in TDT over the age of 25 [19].

The current study excluded pregnant women and individuals with a positive family history of type 1 diabetes to minimize confounding factors. This decision aligns with recent meta-analyses, such as one by Paulo et al., which reported a higher prevalence of diabetes among pregnant women, particularly in Eastern Europe [36]. Additionally, type 1 diabetes is primarily influenced by genetic factors, whereas type 2 diabetes is more often associated with environmental factors, including body weight, diet, and physical activity [15].

Significant increases in GGT levels were observed in TDT patients with impaired glucose metabolism; however, no correlation was found between GGT levels and serum ferritin. This finding suggests that oxidative stress may contribute to the development of DM in TDT patients through mechanisms independent of iron overload. GGT, a well-established marker of oxidative stress, has been associated with an elevated risk of impaired glucose metabolism and type 2 DM [37,38], though its causal role in these conditions remains debated [38].

Some studies, such as one by Fraser et al., have proposed that GGT may be a stronger predictor of diabetes compared to ALT, particularly in non-thalassemia populations [39]. Conversely, research by Abdalla et al., found no significant difference in GGT levels between thalassemia patients and healthy controls, and no correlation between GGT levels and ferritin [40].

Although oxidative stress is not the primary cause of thalassemia, it plays a role in many of its complications. The breakdown of unstable hemoglobin and increased iron burden in TDT patients generate high levels of free radicals. Additional factors such as genetics, lifestyle, comorbidities, and anemia further exacerbate oxidative stress in these patients [41].

A multivariate logistic regression analysis was conducted, incorporating variables such as age, GGT, AST, ALT, heart MRI T2*, and beta-blocker use. However, no significant correlation was identified between these factors and impaired glucose metabolism in TDT patients. This contrasts with findings from Zhang et al., who reported significant associations between serum ferritin, cardiac MRI T2*, and abnormal oral glucose tolerance test (OGTT) results in TDT patients [42].

Furthermore, no statistically significant differences were observed in MRI T2* or serum ferritin levels between splenectomized and non-splenectomized patients. The spleen, a key organ for iron storage (second only to the liver), plays an important role in iron circulation and metabolism, and splenectomy may alter these processes [34,43].

The study identified a statistically significant correlation between heart and pancreatic MRI T2* values, aligning with findings from previous research [17,44]. Hashemieh et al. reported no significant difference in pancreatic iron deposition between diabetic and non-diabetic thalassemia patients when assessed using MRI T2* measurements [45]. Similarly, in the study by Neotzli et al., another index of β-cell function, the disposition index, (DI), was associated with pancreatic R2* MRI values. Interestingly, approximately 30% of patients with increased pancreatic iron overload (measured by R2*) were normoglycemic [10].

The literature suggests that lower pancreatic MRI T2* values are significantly associated with disturbances in glucose metabolism. Cut-off values for pancreatic MRI T2* have been proposed at 5.6 and 17.9 ms, respectively, to identify the risk of impaired glucose metabolism [28,44].

In the study by Meloni et al., a normal pancreatic T2* value was found to be a highly reliable indicator of the absence of cardiac iron overload, with a 100% negative predictive value [17]. Moreover, patients with normal cardiac MRI T2* values showed no evidence of abnormal heart rhythms or heart failure. Conversely, heart MRI T2* values were significantly lower in patients who developed myocardial fibrosis or cardiovascular complications [17,46].

The correlation between heart and pancreatic iron accumulation, as indicated by MRI T2* values, is influenced by patient age and the type of iron chelation therapy used. Notably, the pancreas remains relatively less affected by adipose tissue infiltration, preserving the integrity compared to other organs. In contrast, Pinto et al. did not observe a significant correlation between pancreatic MRI R2* and MRI R2* values from the heart and liver [34]. However, Kosaryan et al. reported a slight correlation between heart and pancreatic MRI T2* values but found no significant correlation between pancreatic iron accumulation and liver iron content [44].

Advancements in cardiac MRI imaging and the development of improved iron chelators have significantly reduced heart failure cases in individuals with TDT. However, older adults with thalassemia continue to report other cardiac complications such as arrhythmias, which can be clinically significant and require regular monitoring. While iron chelation therapy is effective in preventing or treating arrhythmias, some cases may necessitate antiarrhythmic therapy, requiring collaboration with a cardiologist [47].

To our knowledge, no prior studies have examined the impact of beta-blocker use on glucose metabolism disturbances in patients with β-thalassemia. When titrated gradually, beta-blockers are generally well tolerated and effective in managing arrhythmias [7]. The effect of beta-blockers on glucose metabolism varies by type used; for example, metoprolol may negatively affect glucose and lipid control, bisoprolol has a neutral impact on glucose levels, and vasodilating beta-blockers may even improve glycemic control [48].

In our study, a significant correlation was observed between beta-blocker use and the development of impaired glucose metabolism. However, our findings do not establish a clear causal relationship with beta-blockers and it is likely more reflective of disease severity rather than a direct effect of beta-blocker therapy.

This study represents the first attempt to investigate the prevalence and contributing factors of impaired glucose metabolism at the Adult Thalassemia Unit, B Department of Internal Medicine, Hippokration General Hospital. However, several limitations of this retrospective observational study should be noted. OGTT results were available for fewer than half of the patients receiving regular transfusions at the center, despite strong physician recommendations. This underscores the critical need for interdisciplinary collaboration and greater involvement of endocrinologists in the healthcare team.

Similarly, a retrospective study by De Sanctis et al. reported that 65.3% of TDT patients did not comply with the annual OGTT recommendation. Reasons for non-compliance included the inconvenience of frequent visits, intolerance to the glucose load, and hospitalization [3]. According to ICET-A guidelines, diabetic patients with TDT should receive regular evaluations by a multidisciplinary team, including endocrinologists and dietitians with expertise in managing TDT [7].

To better explore the correlation between pancreatic iron overload and glucose metabolism disturbances in a larger population, a prospective observational study should be conducted. This study would involve patients receiving regular transfusions, with all participants undergoing OGTT and pancreatic MRI. Such an approach would enable the identification of any associations between pancreatic iron overload and glucose metabolism disturbances, as well as the tracking of changes in pancreatic iron levels over a defined follow-up period.

Based on previous research conducted at the Adult Thalassemia Unit of Hippokration General Hospital in Thessaloniki, where 140 patients were monitored during the study period, it is estimated that a sample size of 87 subjects would be required to achieve a 95% confidence level with a margin of error of 5%. In the current study, the margin of error was calculated at 7%, which is considered acceptable.

Additionally, it is important to note that MRIs were performed at various diagnostic centers and cardiac echocardiography was conducted by different physicians, which likely contributed to increased diagnostic variability.

## 5. Conclusions

Patients with transfusion-dependent β-thalassemia are at an increased risk of developing impaired glucose metabolism, including diabetes mellitus and impaired glucose tolerance. These conditions result from a combination of insulin resistance and reduced insulin secretion caused by damage to pancreatic β-cells. Our study identified a significant correlation between impaired glucose metabolism and factors such as age, serum GGT levels, and beta-blockers use. Elevated GGT levels in patients highlighted the role of oxidative stress and liver dysfunction in the development of impaired glucose metabolism.

Additionally, pancreas MRI T2* values emerged as a potentially useful predictor of cardiac iron overload in individuals with TDT. Further research is needed to investigate the various contributing factors to glucose metabolism disturbances in TDT patients and to identify reliable biomarkers for predicting glucose regulation. Based on these findings, it is recommended that an OGTT be performed annually to detect early signs of impaired glucose metabolism in this population.

## Figures and Tables

**Table 1:** Estimation of iron overload based on serum ferritin levels and T2* MRI values for heart and liver, SF: serum ferritin.

	SF (ng/mL)	Cardiac MRI T2* (ms)	Hepatic MRI T2* (ms)
Normal	<1000	>20	>8
Mild	1000–2000	14–20	4–8
Moderate	2000–4000	10–14	2–4
Severe	>4000	<10	<2

**Table 2:** Distribution of patients based on glycemic status. The patients were classified into two groups according to their glycemic status: Group 1 included patients without diabetes mellitus (DM) or impaired glucose tolerance (IGT), and Group 2 included patients with DM or IGT.

Groups		
**Group 1**	46/64 (71.8%)	
**Group 2**	18/64 (28.1%)	Diabetes Mellitus: 8/64 (12.5%)
		Impaired Glucose Tolerance: 10/64 (15.6%)

**Table 3:** Descriptive data—comparison between mean values of selected parameters in transfusion-dependent β-thalassemia major patients with and without DM or IGT. Normally distributed numerical variables are expressed as mean value ± standard deviation (mean, SD), while non-normally distributed variables are expressed as median value and interquartile range (median, IQR).

	Normal Distribution: Mean (SD) Non-Normal Distribution: Media (IQR)		*p*-Value
	Group 1	Group 2	
Age	39 (9)	45 (10)	**0.02**
Body weight	65 (18)	72 (20)	0.9
Hemoglobin	9.8 (0.5)	9.8 (0.7)	0.82
Annual transfused Blood volume	10,453 (3336)	9793 (2342)	0.48
WBC	9300 (7900)	12,300 (7770)	0.38
NEU	5258 (2713)	5822 (3675)	0.74
LYM	2896 (1871)	3233 (1843)	0.4
MON	770 (860)	690 (635)	0.9
PLT	421,520 (235,700)	455,100 (237,210)	0.5
GLU	88 (10)	104 (42)	**<0.001**
Urea	39.3 (9)	39.8 (10)	0.93
Serum creatinine	0.8 (0.16)	0.83 (0.2)	0.6
LDH	233 (111)	238 (62)	0.99
Total bil	1.93 (1.17)	1.63 (1.6)	0.34
Direct bil	0.4 (0.32)	0.27 (0.23)	0.63
Indirect bil	1.55 (1.1)	1.3 (1.25)	0.2
GGT	14 (8)	22 (18)	**0.02**
AST	19 (19)	17 (36)	0.6
ALT	21 (13)	20 (17)	0.7
Amylase	54 (24)	45 (28)	0.98
CRP	1.7 (2.8)	1.9 (3.8)	0.4
MRI—liver	11.2 (9.6)	8 (9)	0.7
LIC	2.4 (2.35)	3.3 (4.5)	0.82
MRI—heart	35.3 (5)	33 (7)	0.41
EF	62.3 (5.6)	61.9 (4.5)	0.96
Serum ferritin	720 (506)	620 (620)	0.36

**Table 4:** Comparison of drug administration (hormonal replacement therapy, corticosteroids, beta-blockers, and zinc) between Group 1 and Group 2.

Treatment	YES	NO	
Hormonal replacement therapy	16 (25%)	48 (75%)	0.75
Corticosteroids	2 (3.1%)	62 (96.9%)	0.49
Beta-blocker	13 (20.3%) Group 1:6 Group 2:7	51 (79.7%) Group 1:40 Group 2:11	0.02
Zinc	14 (21.9%)	50 (78.1%)	0.96
n	64	64	

**Table 5:** Comparison of hypothyroidism, HCV infection, and history of splenectomy in Group 1 and Group 2. This table compares the prevalence of hypothyroidism, HCV infection, and history of splenectomy between patients in Group 1 and Group 2. None of these clinical conditions were found to be significantly associated with impaired glucose metabolism (*p* > 0.05).

	Hypothyroidism	HCV Infection	Splenectomy
Group 1	Present: 7 (15.2%) Absent: 39 (84.7%)	Present: 8 (17.3%) Absent: 38 (82.6%)	Positive: 18 (39.1%) Negative: 28 (60.8%)
Group 2	Present: 6 (33.3%) Absent: 12 (66.6%)	Present: 5 (27.7%) Absent: 13 (72.2%)	Positive: 10 (55.5%) Negative: 8 (44.4%)
*p*	0.1	0.35	0.23

**Table 6:** Univariate logistic regression analysis. The table presents the results of the univariate logistic regression analyses for all independent variables. Variables with a *p*-value < 0.2 are highlighted in bold and were subsequently included in the multivariate logistic regression analysis.

Variables (Univariate Logistic Regression Analysis)	*p*-Value
**Age**	**0.03**
Gender	0.63
Blood volume (per year)	0.45
Blood type	0.66
Hemoglobin	0.86
Urea	0.85
Creatinine	0.53
Neutrophils	0.71
Lymphocytes	0.51
Monocytes	0.9
**Glu**	**<0.01**
LDH	0.52
Total bilirubin	0.32
Direct bilirubin	0.62
Indirect bilirubin	0.36
**GGT**	**0.05**
**AST**	**0.11**
**ALT**	**0.11**
Amylase	0.8
CPK	0.21
CRP	0.2
Type of iron chelation	0.33
Liver MRI T2*	0.48
Heart MRI T2*	**0.15**
LIC	0.72
EF	0.76
Hormonal replacement therapy	0.74
**Beta-blocker**	**0.03**
Corticosteroids	0.5
Zinc	0.96
HCV infection	0.35
Splenectomy	0.23
Serum Ferritin	0.43

**Table 7:** Multivariate logistic regression analysis. This table presents the results of the multivariate logistic regression analysis. The analysis shows no statistically significant association between any of the investigated variables and the occurrence of impaired glucose metabolism when other predictors were accounted for. The table includes the odds ratios (OR) and 95% confidence intervals (95% CI) for each variable.

Variables (Multivariate Logistic Regres-Sion Analysis)	*p*-Value	OR	95% CI
Age	0.16	1	0.97–1.1
ALT	0.71	0.98	0.91–1.06
AST	0.64	1	0.96–1.05
GGT	0.14	1	0.99–1.07
Heart MRI T2*	0.19	0.9	0.81–1.04
Beta-blocker	0.18	2.4	0.63–1.7

**Table 8:** Correlation analysis of pancreatic, liver, and heart MRI T2* values, liver iron concentration (LIC), fasting blood glucose, and serum ferritin levels in a subset of 20 β-thalassemia patients.

Pancreatic MRI T2*	Pearson Coefficient	*p*-Value
Liver MRI T2*	−0.27	0.24
Heart MRI T2*	0.45	0.04
LIC	0.16	0.49
Fasting serum glucose (mg/dl)	−0.33	0.15
Serum ferritin (μg/L)	−0.05	0.83

## Data Availability

The data supporting the findings of this study are available from the corresponding author upon reasonable request.
